# Fear of Falling in Women with Fibromyalgia and Its Relation with Number of Falls and Balance Performance

**DOI:** 10.1155/2015/589014

**Published:** 2015-11-05

**Authors:** D. Collado-Mateo, J. M. Gallego-Diaz, J. C. Adsuar, F. J. Domínguez-Muñoz, P. R. Olivares, N. Gusi

**Affiliations:** ^1^Faculty of Sport Sciences, University of Extremadura, Avenida Universidad S/N, 10003 Cáceres, Spain; ^2^Instituto de Actividad Física y Salud, Universidad Autónoma de Chile, 5 Poniente 1670, 3460000 Talca, Chile

## Abstract

*Objective*. To evaluate fear of falling, number of falls, and balance performance in women with FM and to examine the relationship between these variables and others, such as balance performance, quality of life, age, pain, and impact of fibromyalgia. *Methods*. A total of 240 women participated in this cross-sectional study. Of these, 125 had fibromyalgia. Several variables were assessed: age, fear of falling from 0 to 100, number of falls, body composition, balance performance, lower limb strength, health-related quality of life, and impact of fibromyalgia. *Results*. Women with fibromyalgia reported more falls and more fear of falling. Fear of falling was associated with number of falls in the last year, stiffness, perceived balance problems, impact of FM, and HRQoL whereas the number of falls was related to fear of falling, balance performance with eyes closed, pain, tenderness to touch level, anxiety, self-reported balance problems, impact of FM, and HRQoL. *Conclusion*. FM has an impact on fear of falling, balance performance, and number of falls. Perceived balance problems seem to be more closely associated with fear of falling than objective balance performance.

## 1. Introduction

Fibromyalgia (FM) is a chronic disease found primarily in women. It is characterized by widespread pain and several associated symptoms, such as nonrestorative sleep, fatigue, poor physical conditioning, impaired cognition, stiffness, depression, and balance impairment [1, 2]. These symptoms often lead to a reduction in health-related quality of life (HRQoL) [3] and hinder the ability to perform activities of daily living (ADL) [4]. Although the causes of FM are still unknown, the up-to-date most accepted hypothesis is the sensitization of the central nervous system [5], which proposes that the cause of the high level of pain is the amplification of the sensory inputs by the central nervous system. The estimated overall prevalence of FM oscillates from 2.9% to 4.7% in the general population [6]. FM imposes significant economic burden caused, among other reasons, by the high prevalence of work loss [7, 8].

Previous studies have demonstrated reduced postural stability and increased frequency of falls in FM patients and have emphasized the need to understand the factors and characteristics that could be associated with them [9–11]. There is no consensus on the fall predictors, and the relationship between fear of falling, frequency of falls, and other factors such as age, level of pain, fatigue, HRQoL, or balance impairment is not clear. In this regard, it has been hypothesized that fall status is predicted by perception of postural instability, balance performance, and executive function processing speed [12], impact of FM measured by the Fibromyalgia Impact Questionnaire (FIQ) [10, 13], hip extension rate of torque development, duration of fibromyalgia symptoms, overall pain, and knee pain [13]. Similarly, balance performance has been associated with strength, pain [13], sleep quality, and fatigue [14]. Most of these studies stated that there is a need for further studies with larger samples. To the best of our knowledge, there is no study focused on the assessment of balance and fall status with a sample higher than 70 women with FM and 70 controls.

FM is associated with high prevalence of overweight and obesity [15]. Physical inactivity is a common characteristic in women with FM and may cause 72% of this population to be overweight [16]. This sedentary tendency could be a consequence of the large number of symptoms associated with the disease, but Rutledge et al. [17] observed that fear of falling often makes women with FM unable to continue with their usual physical activities, especially those who have fallen recently. Fear of falling may limit the ability to perform ADL and physical exercise, but there is lack of studies on the fear of falling and its relation with balance performance, impact of FM, age, pain, weight, and other symptoms of FM.

The current study has two objectives: the first goal was to assess balance performance, fear of falling, and frequency of falls in women with FM and to compare these results with those from women without FM. The second objective was to examine the relationship between fear of falling, number of falls, and other variables, such as pain, impact of FM, age, body mass index (BMI), HRQoL, and FM-associated symptoms.

## 2. Methods

### 2.1. Participants

A total of 240 women participated in the study. Of these 240 participants, 125 were women diagnosed with FM, and 115 were women without FM. Participants were recruited at local FM associations, community associations, and the University of Extremadura, including the University for the Elderly.

Inclusion criteria were set as follows: (a) being a woman diagnosed with FM by a rheumatologist according to the criteria of the American College of Rheumatology [18], (b) being able to communicate effectively with the study staff, and (c) reading and signing the written informed consent. Participants were excluded if they (a) are not able to stand by themselves, (b) have severe visual or hearing impairment, and (c) have vestibular diseases. This study was approved by the Committee of Bioethics of the University of Extremadura (Spain). It was developed in accordance with the Spanish legislation on bioethics, biomedical research, and personal data confidentiality, and it satisfied the values of the updated Helsinki Declaration.

### 2.2. Procedure

All participants came to the association's building or to the laboratory at the university. They were informed and signed the written informed consent. The protocol started with the body composition analysis using Tanita body composition analyzer BC-418 MA. After that, they were asked to complete 2 physical tests, balance performance and lower limb muscle strength tests, and finally, participants completed the questionnaires.

The first physical test was the Clinical Test of Sensory Integration of Balance (CTSIB). It is a balance protocol with more than 25 references in PubMed (Medline). It was conducted using the Biodex Balance System (Shirley, NY, USA). The CTSIB test comprises 4 conditions: eyes open on firm surface, eyes closed on firm surface, eyes open on unstable surface, and eyes closed on unstable surface. In all tests, patients had to maintain their feet on the platform for 30 seconds and had to rest for 10 seconds between each test. Feet position was controlled using adhesive footprint marks on the balance platform. The position of these footprints was based on the study by McIlroy and Maki [19] who found that the most comfortable foot position for women was a heel-to-heel distance of 16 cm and an external rotation of 15°. The sway index was used for the analysis. This index quantifies how much the person swayed over the 30 seconds and is calculated as the standard deviation of the sway angle [20].

The second physical test was the 30 s chair stand test. This test was performed after the previous test, with a rest of 5 minutes. Participants had to start seated on a chair with their hands over their shoulders. They had to stand up and sit down as fast as possible within 30 seconds [21]. The number of times they were able to stand up was recorded.

Finally, participants were asked to complete the EQ-5D-5L [22], a single question about the number of falls in the last six months, another one about the number of falls in the last year, and a Visual Analog Scale (VAS) where women had to report their fear of falling from 0 (no fear) to 100 (extreme fear). Additionally, women with FM completed the Fibromyalgia Impact Questionnaire (FIQ) [23] and its revised version (FIQ-R) [24].

The EQ-5D-5L is a widely used preference-based HRQoL questionnaire [22] that consists of 5 dimensions (mobility, self-care, usual activities, pain or discomfort, and anxiety or depression), with five possible levels of problem. It includes a VAS to evaluate the perceived health status from 0 (worst imaginable health status) to 100 (best imaginable health status). Therefore, the current study used 2 different VAS: (a) the VAS assessing fear of falling from 0 to 100 and (b) the EQ-5D-VAS assessing health status from 0 to 100.

The FIQ [23] is a 10-item instrument with three domains: function, overall impact, and symptoms. In this study, the consensus version for Spanish population developed by Esteve-Vives et al. [25] was used. The FIQ was revised and modified in 2009 [24]. The validation of the Spanish version of FIQ-R was developed by Salgueiro et al. [26].

### 2.3. Statistical Analysis

Statistical analysis was performed using SPSS software (Windows version 21.0, Chicago, Illinois, USA). Between-group differences were calculated using Student's *t*-test. This test was conducted for the whole sample, that is, 125 women with FM and 115 women without FM. Given that normal age-related changes often lead to deterioration of the physical conditioning and health, the sample was divided into three age groups (less than 50 years, 50–59 years, and more than 60 years). A multivariate analysis of variance (MANOVA) was performed, with a 3 (age groups) × 2 (with and without FM) factorial design. Partial eta-squared (*ηp* ^2^) was calculated to gauge the magnitude of the differences. Correlation analyses were used to evaluate the relationship between number of falls, fear of falling, and the rest of the variables: body composition, balance performance, age, FIQ score, FIQ-R score, EQ-5D-5L index, and health VAS in the fibromyalgia group, by Pearson correlation test (*r*) values. FIQ-R dimensions were also included in this analysis. The level of significance was set at *p* < .05.

## 3. Results

Table 1 summarizes the differences between women with and without FM. There were significant differences in all variables, except in body composition and BMI. Participants with FM had higher scores in the 4 tasks of the balance test, which means poorer balance control. Fear of falling was 33% higher in women with FM compared with participants without FM. There were significant differences in the number of falls in the last year and in the last 6 months. Women suffering from FM reported more than 3 times the number of falls of women without FM. As expected, HRQoL and perceived health status from 0 to 100 were poorer in women with FM.

Table 2 shows the effect of having or not FM, age, and the interaction of both measures on the key variables. Regarding the group effect, results were consistent with those previously reported in Table 1. The magnitude of differences was small for fear of falling and balance with eyes open on firm surface; moderate for number of falls, balance with eyes closed, and balance on unstable surface; and large for health status, strength, and HRQoL assessed using EQ-5D-5L. The criteria for determining the magnitude of *ηp* ^2^ was the following: 0.01–0.06 = small, 0.06–0.14 = moderate, and >0.14 = large [27].

Age had a significant effect on BMI, fat mass, muscular mass, fear of falling, balance on unstable surface, and strength. The magnitude of differences was moderate for fat mass, muscular mass, fear of falling, balance with eyes closed on unstable surface, and strength, whereas it was small for BMI and balance with eyes open on unstable surface. The interaction of group (with or without FM) and age was not significant in any of the assessed variables.

**Figure 1:**
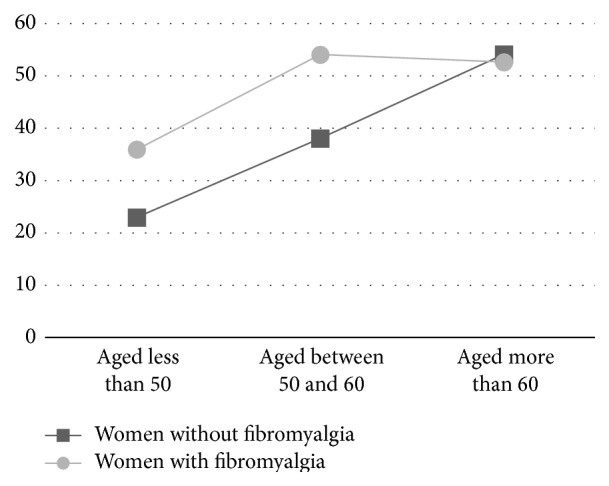
Fear of falling of women with and without fibromyalgia.

In the non-FM group, the self-reported fear of falling was increased as a consequence of age. In this regard, women without FM aged less than 50 years reported fear of falling of 22.97 (25.69), whereas women aged between 50 and 59 reported fear of falling of 38.07 (37.59), and those older than 60 reported fear of falling of 54.14 (32.60). As can be observed in Figure 1, there is an expected linear increase. However, in the FM groups, this tendency is not observed. The younger group reported fear of falling of 35.93 (34.72), and the other two groups reported mean fear of falling of 54.09 and 52.65, respectively.

The relationship between fear of falling, number of falls, and the other analyzed variables is displayed in Table 3. This table was generated with data from all women with FM who participated in the study. Fear of falling was significantly related to the number of falls in the last year, stiffness (measured by FIQ-R), and perceived balance impairment (measured by FIQ-R). Other variables such as age, BMI, muscle mass, function (measured by FIQ-R), and FIQ-R total score were near to be significant (*p* < .1) but were considered nonsignificant. The number of falls in the last year was significantly associated with fear of falling, balance performance with eyes closed on firm and unstable surface, pain, tenderness to touch level, self-reported balance problems, impact of FM, and HRQoL. On the other hand, the number of falls in the last 6 months was related to self-reported balance problems, anxiety, impact of FM, and HRQoL.

## 4. Discussion

The main finding of the current paper was that there are important differences in fear of falling and number of falls between women with and without FM. As can be observed in Figure 1, the evolution on fear of falling is different between groups. Women without FM experienced fear of falling that is gradually increased as the age is increased. On the other hand, women with FM start with higher levels of fear, which are rapidly increased and then maintained. In fact, fear of falling was not significantly related to age in women with FM. In this regard, fear of falling was only significantly associated with the number of falls in the last year, stiffness, and perceived balance problems. There was no significant association between depression assessed using FIQ-R and fear of falling, supporting findings from a previous study in adults [28]. To our knowledge, this is the first study that aims to examine the variables associated with fear of falling in women with FM.

The MANOVA in Table 2 shows no significant effect of the group-age interaction. This could mean that women with and without FM are similarly affected by age. Regarding fear of falling, Student's *t*-test analysis showed that women with FM aged between 50 and 59 had significantly higher levels of fear of falling than women without FM at the same age, whereas there were no statistically significant differences in the other 2 age groups.

The current study supports findings from the previous study by Rutledge et al. [12], who observed that perception of postural instability, balance performance, and executive function processing speed were predictors of falling status. In the current paper, the number of falls in the last year was related to perceived balance problems and balance performance in tasks with eyes closed. Additionally, Table 3 shows that self-reported level of pain, tenderness to pain, anxiety (all three measured by FIQ-R), impact of FM, and EQ-5D-5L index may be related to the number of falls.

Balance performance in tasks with eyes closed seems to be more associated with fall status than performance on tasks where the eyes are open. Interestingly, between-group differences in tasks with eyes closed and/or unstable surface are higher than those differences observed in the task with eyes open on stable surface. Therefore, an important implication of these results may be the relevance of training balance performance with eyes closed. In this regard, few recent studies have suggested that balance exercise should be included in comprehensive programs [29, 30]. However, according to our results, balance exercise should consider training with the eyes closed.

In the scientific literature, there are several articles reporting worst balance among women with FM compared with healthy subjects. The results of the current study support that notion because statistically significant differences in all 4 balance tasks (*p* < .001) can be observed. The role of pain in balance has been previously studied. Among other findings, Sipko and Kuczyński [31] showed that those persons with high levels of pain relied more on visual input than those with low pain. In the current study, there was a significant correlation between the performance in the balance tasks with the eyes closed and the number of falls. Therefore, the need of adding motor control activities with eyes closed is reinforced.

The number of falls was gradually increased as a consequence of age in the FM group. On the other hand, this increment was not observed in women without FM. Women aged less than 50 years and without FM fell 0.60 (1.50) times in the last year, whereas those aged 50–59 years fell 0.23 (0.60) and the older group fell 0.38 times. Talbot et al. [32] observed a similar percentage (near 20%) of fallers in a group of 292 young adults (aged 20–45 years) and in 616 middle-aged adults, including males and females. The observed high prevalence of falls among women aged less than 50 may be a consequence of the higher activity level of younger adults. Additionally, it could be higher because the selected cutoff was higher than the selected one in the cited article.

One of the main strengths of the current paper is that balance was objectively and subjectively assessed. First, participants completed the balance tasks, and after that, they were asked about their perceived balance problems. In this regard, fear of falling was only significantly associated with perceived balance problems and not with the scores in the tasks. On the other hand, the number of falls was significantly related to both objective balance performance and self-reported balance problems. Given that fear of falling is a subjective feeling, it seems plausible that perceived balance problems are more important than the actual balance performance.

Different clinical implications can be stated from the results of the current study. First, there is a need of including balance tasks with the eyes closed in physical exercise interventions. These interventions should also consider the perceived balance problems of women with FM, as this perception is associated with fear of falling and number of falls. Second, given the wide range of variables associated with fear of falling and number of falls, treatment of FM should be done from a multidisciplinary approach, including pharmacological and nonpharmacological therapies.

The current paper has 4 main limitations. Although to our knowledge this paper has the largest sample of the studies focused on measuring objective balance performance, number of falls, and fall risk in women with FM, 240 participants could be not enough to observe and ensure all the differences when analysis is performed for three age groups. The second limitation could be that in the current paper there was not any instrument that evaluates physical activity level. Similarly, the treatments (both pharmacological and nonpharmacological) of women were not controlled. The fourth limitation is the uncontrolled comorbidities. In this regard, exclusion criteria include vestibular diseases and hearing and visual impairments. However, several conditions that could be suffered together with FM, like depression, diabetes, and obesity, among others, could affect the results. Despite these 4 limitations, the current study provides relevant information about balance performance, falls, and fear of falling and contributes to the understanding of these variables in women suffering from FM.

## 5. Conclusion

FM has an important impact on balance performance, number of falls, and fear of falling. There are important differences in fear of falling and number of falls between women with and without FM. Fear of falling in women with FM was associated with number of falls in the last year, stiffness, perceived balance problems, impact of FM, and HRQoL whereas number of falls was related to fear of falling, objective balance performance with eyes closed on firm and unstable surface, pain, tenderness to touch level, anxiety, self-reported balance problems, impact of FM, and HRQoL.

## Figures and Tables

**Table 1:** Differences between women with and without FM.

	Fibromyalgia (*n* = 125)	Not fibromyalgia (*n* = 115)	*p* value
Age (years)	55.42 (10.35)	54.23 (10.68)	.381

BMI (kg/m^2^)	25.59 (4.06)	25.30 (3.58)	.569
Muscular mass (%)	62.35 (7.29)	62.30 (5.87)	.953
Fat mass (%)	34.35 (7.10)	34.45 (6.18)	.911

Duration of symptoms (years)	21.33 (12.19)	NA	NA
Years since diagnosis	10.87 (7.13)	NA	NA

Balance EOFS (sway index)	0.74 (0.49)	0.58 (0.22)	<.001
Balance ECFS (sway index)	1.19 (0.87)	0.80 (0.32)	<.001
Balance EOUS (sway index)	1.32 (0.58)	1.02 (0.32)	<.001
Balance ECUS (sway index)	3.10 (0.96)	2.61 (0.72)	<.001

Fear of falling (0–100)	48.88 (33.84)	36.61 (34.33)	.006

Number of falls in the last year	1.45 (2.49)	0.40 (1.05)	<.001
Number of falls in the last 6 months	0.80 (1.52)	0.18 (0.60)	<.001

Strength (number of repetitions)	10.04 (2.26)	12.47 (2.56)	<.001

EQ-5D-5L	0.52 (0.24)	0.95 (0.08)	<.001
Health VAS	49.47 (23.62)	84.44 (14.40)	<.001

NA: not available; EOFS: eyes open on firm surface; ECFS: eyes closed on firm surface; EOUS: eyes open on unstable surface; ECUS: eyes closed on unstable surface; VAS: Visual Analog Scale.

**Table 2:** Effects of group (with or without FM) and age on key measures.

	Age group	Healthy controls		Women with fibromyalgia		Group effect		Age effect		Interaction Group *∗* age	
		Mean	SD	Mean	SD	*p* value	Partial eta-squared	*p* value	Partial eta-squared	*p* value	Partial eta-squared
BMI	<50	24.31	3.48	24.03	4.09	.704	.001	.001	.058	.541	.005
	50–59	25.81	3.81	25.64	3.71						
	>60	26.04	3.20	27.08	4.34						

Fat mass	<50	32.52	6.06	30.96	8.40	.636	.001	<.001	.072	.520	.006
	50–59	35.60	6.20	34.89	5.74						
	>60	35.58	5.93	36.60	7.41						

Muscular mass	<50	64.12	5.75	65.92	8.75	.637	.001	<.001	.072	.488	.006
	50–59	61.21	5.88	61.56	6.13						
	>60	61.24	5.65	60.33	7.15						

Health VAS	<50	82.50	14.92	45.32	21.98	<.001	.454	.125	.018	.848	.001
	50–59	87.27	12.97	52.69	24.20						
	>60	83.55	15.51	50.00	20.60						

Number of falls in the last year	<50	0.60	1.50	1.23	2.60	<.001	.066	.602	.005	.493	.006
	50–59	0.23	0.61	1.31	2.26						
	>60	0.38	0.73	1.81	2.88						

Number of falls in the last 6 months	<50	0.31	0.84	0.90	1.90	<.001	.061	.580	.005	.777	.002
	50–59	0.05	0.21	0.80	1.53						
	>60	0.21	0.56	0.69	1.15						

Fear of falling	<50	22.98	25.69	36.77	34.97	.042	.018	<.001	.075	.181	.015
	50–59	38.95	37.57	55.09	33.79						
	>60	54.14	32.60	51.25	29.70						

Balance EOFS (sway index)	<50	0.53	0.21	0.75	0.57	.007	.031	.866	.001	.193	.015
	50–59	0.57	0.18	0.77	0.53						
	>60	0.67	0.27	0.67	0.27						

Balance ECFS (sway index)	<50	0.81	0.39	1.33	1.08	<.001	.078	.463	.007	.374	.009
	50–59	0.79	0.26	1.23	0.95						
	>60	0.82	0.32	1.03	0.43						

Balance EOUS (sway index)	<50	0.87	0.21	1.26	0.73	<.001	.087	.002	.055	.099	.020
	50–59	0.99	0.23	1.31	0.49						
	>60	1.29	0.39	1.37	0.36						

Balance ECUS (sway index)	<50	2.24	0.39	2.77	0.98	<.001	.064	<.001	.090	.678	.003
	50–59	2.70	0.82	3.15	0.89						
	>60	3.00	0.70	3.29	0.90						

Strength (number of repetitions)	<50	13.63	2.58	10.56	2.56	<.001	.201	<.001	.074	.235	.013
	50–59	11.92	1.97	10.00	1.91						
	>60	11.48	2.67	9.48	2.26						

EQ-5D-5L utility index	<50	0.97	0.06	0.49	0.24	<.001	.606	.308	.010	.214	.014
	50–59	0.95	0.10	0.56	0.22						
	>60	0.92	0.08	0.50	0.22						

EOFS: eyes open on firm surface; ECFS: eyes closed on firm surface; EOUS: eyes open on unstable surface; ECUS: eyes closed on unstable surface; VAS: Visual Analog Scale.

**Table 3:** Relationship between fear of falling, number of falls, and the other analyzed variables.

	*N*	Fear of falling		Number of falls in the last year		Number of falls in the last 6 months	
		*R*	*p* value	*R*	*p* value	*R*	*p* value
Fear of falling	121			.178^*∗*^	.049	.117	.205

Number of falls in the last year	121	.178^*∗*^	.049			.886^*∗∗*^	<.001
Number of falls in the last 6 months	121	.117	.205	.886^*∗∗*^	<.001		

Balance EOFS (sway index)	121	−.085	.351	.044	.632	.042	.647
Balance ECFS (sway index)	121	.037	.685	.196^*∗*^	.031	.166	.071
Balance EOUS (sway index)	121	−.049	.595	.070	.446	.038	.681
Balance ECUS (sway index)	121	−.010	.917	.199^*∗*^	.029	.103	.264

Strength (number of repetitions)	121	−.159	.082	−.008	.933	.018	.849

Age (years)	121	.161	.078	.046	.620	−.080	.384

BMI (kg/m^2^)	121	.157	.085	.031	.732	.011	.902
Fat mass (%)	119	.147	.111	.054	.559	.049	.601
Muscular mass (%)	119	−.155	.092	−.048	.605	−.043	.641

FIQ-R score	113	.160	.090	.178	.059	.227^*∗*^	.016
FIQ-R functional domain	114	.169	.073	.130	.167	.181	.055
FIQ-R overall domain	113	.048	.611	.055	.562	.127	.181
Pain	113	.018	.853	.211^*∗*^	.025	.182	.055
Energy	111	.136	.155	.006	.952	.023	.816
Stiffness	112	.189^*∗*^	.046	.175	.065	.163	.088
Sleep quality	112	.018	.850	.141	.139	.158	.097
Depression	112	.145	.128	.098	.302	.074	.443
Memory problems	113	.118	.212	.084	.374	.137	.151
Anxiety	112	.136	.153	.179	.059	.221^*∗*^	.020
Tenderness to pain	113	.085	.371	.201^*∗*^	.033	.181	.056
Balance problems	113	.213^*∗*^	.023	.332^*∗∗*^	<.001	.297^*∗∗*^	.001
Sensitivity to loud noises, bright lights, odors, and cold	113	.041	.670	.136	.150	.139	.144

FIQ score	114	.126	.181	.216^*∗*^	.021	.294^*∗∗*^	.002

EQ-5D-5L index	120	−.149	.103	−.218^*∗*^	.017	−.239^*∗∗*^	.009
Health VAS	121	−.010	.912	−.007	.939	−.071	.443

EOFS: eyes open on firm surface; ECFS: eyes closed on firm surface; EOUS: eyes open on unstable surface; ECUS: eyes closed on unstable surface; FIQ-R: Revised Fibromyalgia Impact Questionnaire; FIQ: Fibromyalgia Impact Questionnaire; VAS: Visual Analog Scale. ^*∗*^ *p* < .05; ^*∗∗*^ *p* < .01.
